# A microbial causal mediation analytic tool for health disparity and applications in body mass index

**DOI:** 10.21203/rs.3.rs-2463503/v1

**Published:** 2023-01-13

**Authors:** Chan Wang, Jiyoung Ahn, Thaddeus Tarpey, Stella S. Yi, Richard B. Hayes, Huilin Li

**Affiliations:** 1 Division of Biostatistics, Department of Population Health, New York University Grossman School of Medicine, New York, 10016, NY, USA; 2 Division of Epidemiology, Department of Population Health, New York University Grossman School of Medicine, New York, 10016, NY, USA; 3 Department of Population Health Section for Health Equity, New York University Grossman School of Medicine, New York, 10016, USA

**Keywords:** Casual mediation model, Health disparity, Manipulable disparity measure, Microbiome mediator, Non-manipulable exposure

## Abstract

**Background:**

Emerging evidence suggests the potential mediating role of microbiome in health disparities. However, no analytic framework is available to analyze microbiome as a mediator between health disparity and clinical outcome, due to the unique structure of microbiome data, including high dimensionality, sparsity, and compositionality.

**Methods:**

Considering the modifiable and quantitative features of microbiome, we propose a microbial causal mediation model framework, SparseMCMM_HD, to uncover the mediating role of microbiome in health disparities, by depicting a plausible path from a non-manipulable exposure (e.g. race or region) to a continuous outcome through microbiome. The proposed SparseMCMM_HD rigorously defines and quantifies the manipulable disparity measure that would be eliminated by equalizing microbiome profiles between comparison and reference groups. Moreover, two tests checking the impact of microbiome on health disparity are proposed.

**Results:**

Through three body mass index (BMI) studies selected from the curatedMetagenomicData 3.4.2 package and the American gut project: China vs. USA, China vs. UK, and Asian or Pacific Islander (API) vs. Caucasian, we exhibit the utility of the proposed SparseMCMM_HD framework for investigating microbiome’s contributions in health disparities. Specifically, BMI exhibits disparities and microbial community diversities are significantly distinctive between the reference and comparison groups in all three applications. By employing SparseMCMM_HD, we illustrate that microbiome plays a crucial role in explaining the disparities in BMI between races or regions. 11.99%, 12.90%, and 7.4% of the overall disparity in BMI in China-USA, China-UK, and API-Caucasian comparisons, respectively, would be eliminated if the between-group microbiome profiles were equalized; and 15, 21, and 12 species are identified to play the mediating role respectively.

**Conclusions:**

The proposed SparseMCMM_HD is an effective and validated tool to elucidate the mediating role of microbiome in health disparity. Three BMI applications shed light on the utility of microbiome in reducing BMI disparity by manipulating microbial profiles.

## Background

Health disparities refer to the inequalities in the quality of health, health care, and health outcomes experienced by groups that are usually classified by race, ethnicity, and region. Many factors, including genetics, social-economic status, culture, dietary habits, and geographical conditions, contribute to health disparities between groups. Researchers have long been interested in identifying the modifiable environmental determinants of health disparity to pave the way to improve health equity. However, environmental exposures are often numerous, ubiquitous, descriptive, or hard to measure, which makes this task difficult.

**Figure 1.**
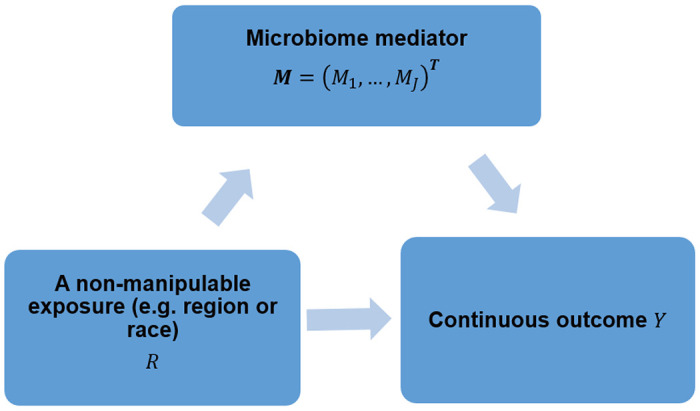
Microbiome (*M*) may play a mediating role in the health disparity of the continuous outcome (*Y*) between two categories of a non-manipulable exposure group (e.g. race or region) (*R*). We aim to investigate how much disparity of the outcome *Y* can be reduced by manipulating microbiome profiles.

Gut microbiome is the aggregate of all genomes harbored by gut microbiota, which is the collection of all microbes that reside in human gut. Benefiting from the advent of high throughput sequencing technologies, a great number of microbiome studies have been conducted to quantitatively characterize the microbiome profiling and understand its role in human health [1–4]. Gut microbiome has been closely linked with host metabolic, immune, and neuroendocrine functions [5–12]. On the other hand, many environmental and social factors, such as diet, drugs, lifestyle, psychological state and behavior, aid in shaping gut microbial profiles [13–16]. Recently, the mediating role of microbiome between these environmental exposures and various human diseases, including obesity, type 2 diabetes, inflammatory bowel disease, depression, and different cancers, has been investigated and recognized [17–22]. Given the modifiable and quantitative features of microbiome, we here aim to disentangle health disparities by exploring the extent of the observed disparity in the outcome of interest that could be reduced if the gut microbial profile was modified. In Figure 1, we propose a mediation framework to answer such questions. Here, the disparity group, e.g., race or region, is the exposure denoted by *R*; the gut microbial profile is the mediator denoted by *M*; and the continuous study outcome, e.g., body mass index (BMI), is denoted by *Y*.

There are several existing mediation analysis frameworks tailored for non-manipulable exposures, such as race, region, sex or socioeconomic position [27], however, due to the unique structure of microbiome data, including high dimensionality, sparsity and compositionality, these approaches are not immediately applicable for analyzing microbiome as a mediator for health disparity. Recently, we developed a rigorous Sparse Microbial Causal Mediation Model (SparseMCMM) [12] for interrogating the mediating role of microbiome in a typical three-factor (randomized treatments, microbiome as mediator, and outcome) clinical trial causal study design. SparseMCMM quantifies the overall mediation effect of microbiome community and the component-wise mediation effect for each individual microbe under the counterfactual framework, identifies the signature causal microbes with regularization strategies, and tests the mediation effects while fully acknowledging the unique structure of microbiome data. In this paper, by extending SparseMCMM to a non-manipulable exposure setting, we propose a microbial causal mediation framework for health disparity study and denote it as SparseMCMM_HD (SparseMCMM for Health Disparity). As VanderWeele and Robinson [23] discussed, causal interpretation of a non-manipulable exposure, i.e., ethnicity or region, is not definable in the traditional counterfactual framework, because a hypothetical intervention on a non-manipulable exposure is not possible. Instead, one can interpret the causality of health inequality by the hypothesized intervention effect on the manipulable mediating variable. Thus, in SparseMCMM_HD, we aim to quantify the overall health inequality on the outcome (called overall disparity), the health inequality effect that would be eliminated by equalizing microbiome profiles across racial or regional groups (called manipulable disparity), and the healthy inequality effect that would remain even after microbiome profiles across racial or regional groups were equalized (called residual disparity). In addition, we equip two hypothesis tests to examine the mediating role of microbiome in health disparity and statistically identify which specific microbes contribute to it.

Obesity (defined via BMI) is a global epidemic and a persistent public health problem [24]. It is well documented that the prevalence of adult obesity is distributed unevenly across racial groups and regions. Partial effect of manipulable exposures such as diet, medication, and antibiotics use [17–19] on obesity has been shown to be mediated through microbiome. In addition, accumulating evidence indicates that gut microbial profile varies across ethnicities as well as geographically [25–27]. Together, these studies suggest that microbiome may play a mediating role in the ethnic or regional disparity of obesity. It is crucial to investigate rigorously how much health inequalities in BMI can be reduced by manipulating microbiome profiles. Utilizing SparseMCMM_HD, we investigate the role of microbiome in the regional and racial disparity of BMI in curated microbiome data from the curatedMetagenomicData 3.4.2 package [28] and the American Gut Project (AGP) (www.americangut.org) respectively. Through these real data analyses, we illustrate a clear and plausible causal path analysis to understand the current racial or regional disparity in BMI and identify a comprehensive set of mediating microbial taxa. The proposed analytic pipeline is available through an interactive web app at https://chanw0.shinyapps.io/sparsemcmm_hd/. We believe that this novel pipeline will be useful for investigating the manipulable disparity through gut microbiome and understanding the causes of health disparity.

## Methods

### SparseMCMM_HD framework

#### Casual mediation model.

Suppose there are *I* subjects from two categories of a non-manipulable exposure group (e.g. race or region), *J* taxa, and *K* covariates. Subscripts *i, j, k*, indicate a subject, a taxon, and a covariate respectively. For the *i*th subject, let *R*_*i*_ = 1 or 0 indicate the reference or comparison group, let $M_{i}={\left(M_{i1},\ldots ,M_{iJ}\right)}^{T}$ be the microbiome relative abundance vector with the constraint $\sum_{j=1}^{J}M_{ij}=1$, and let $X_{i}={\left(X_{i1},\ldots ,X_{iK}\right)}^{T}$ represent the covariates, and let *Y_i_* be a continuous outcome of interest.

To statistically describe the causal relationships shown in Figure 1, following our previous work [12], we use the linear log-contrast model to regress the continuous outcome on the non-manipulable exposure, microbiome compositions, interactions between the non-manipulable exposure and microbiome compositions, while adjusting the confounding covariates:

(1) $$\begin{array}{l} Y_{i}=\alpha_{0}+\alpha_{X}^{T}X_{i}+\alpha_{R}R_{i}+\alpha_{M}^{T}\left[\log\left(M_{i}\right)\right]+\alpha_{C}^{T}\left[\log\left(M_{i}\right)\right]R_{i}+\epsilon_{i},\\ \;\;\text{subject to}\;\alpha_{M}^{T}1=0,\text{and}\;\alpha_{C}^{T}1=0, \end{array}$$

where *α*_0_ is the intercept, *α*_*R*_ is the coefficient of the non-manipulable exposure, $\alpha_{X}={\left(\alpha_{X1},\ldots ,\alpha_{XK}\right)}^{T},\alpha_{M}={\left(\alpha_{M1},\ldots ,\alpha_{MJ}\right)}^{T}$, and $\alpha_{C}={\left(\alpha_{C1},\ldots ,\alpha_{CJ}\right)}^{T}$ are the vectors of coefficients of covariates, microbiome compositions, interactions between the non-manipulable exposure and microbiome compositions, respectively. Due to the compositionality of microbiome data as $\sum_{j=1}^{J}M_{ij}=1,\alpha_{M}$ and $\alpha_{{}_{C}}$ are subject to $\alpha_{M}^{T}1=0$ and $\alpha_{C}^{T}1=0.\epsilon_{i}∼N\left(0,\sigma^{2}\right)$ is the error term. On the other hand, the Dirichlet regression [29] is used to model the microbial relative abundance as a function of the non-manipulable exposure and covariates:

(2) $$\begin{array}{c} E\left[M_{ij}\right]=\frac{\gamma_{j}\left(R_{i},X_{i}\right)}{\sum_{m=1}^{J}\gamma_{m}\left(R_{i},X_{i}\right)},\\ \log\left\{\gamma_{j}\left(R_{i},X_{i}\right)\right\}=\beta_{0j}+\beta_{Rj}R_{i}+\beta_{Xj}^{T}X_{i}. \end{array}$$

Specifically, we assume that $M_{i}|\left(R_{i},X_{i}\right)∼\text{Dirichlet}\left(\gamma_{1}\left(R_{i},X_{i}\right),\ldots ,\gamma_{J}\left(R_{i},X_{i}\right)\right)$, and their microbial relative means are linked with the non-manipulable exposure and covariates (*R*, ***X***_*i*_) in the generalized linear model fashion with a log link. $\beta_{0j}$ is the intercept and *β*_*Rj*_ and ***β***_*Xj*_ are the coefficients of the non-manipulable exposure and covariates for the *j*th taxon, respectively.

#### Definition of disparity measures in the counterfactual framework.

As discussed in the Background, we propose to conceptualize an overall disparity measure (ODM) on the outcome that can be decomposed into manipulable disparity measure (MDM) and residual disparity measure (RDM). MDM represents the portion of disparity that would be eliminated by equalizing microbiome profiles between comparison and reference groups, and RDM represents the portion that would remain even after microbiome profiles between comparison and reference groups were equalized. With the counterfactual notation, mathematically we have:

$$\begin{array}{l} \;\text{ODM}=\text{MDM}+\text{RDM},\\ \text{MDM}=E\left[E\left[Y_{M_{x}(1)}|R=1,x\right]\right]-E\left[E\left[Y_{M_{x}(0)}|R=1,x\right]\right],\text{and}\\ \text{RDM}=E\left[E\left[Y_{M_{x}(0)}|R=1,x\right]-E\left[Y_{M_{x}(0)}|R=0,x\right]\right]. \end{array}$$

Here, ***M***_*x*_(0) (***M***_*x*_(1)) is a random value from the microbiome distribution of the reference (comparison) population with given covariates ***x***. *Y*_*m*_ denotes an individual’s potential counterfactual outcome if his or her microbial mediators were set to ***m***, where ***m*** can be $M_{x}(0)\;\text{or}\;M_{x}(1).E\left[Y_{M_{x}(0)}|R=0,x\right]\left(E\left[Y_{M_{x}(1)}|R=1,x\right]\right)$ denotes the expected outcome for a reference (comparison) individual with given covariates $x,E\left[Y_{M_{x}(0)}|R=1,x\right]$ denotes the expected outcome for a comparison individual with given covariates ***x*** if their microbial mediators were set to a random value from that of the reference population with the same covariates ***x***.

#### MDM, RDM, and ODM expressions.

Two assumptions must be satisfied for the identification of MDM, RDM, and ODM [23, 30]. The effect of non-manipulable exposure *R* on outcome *Y* are unconfounded conditional on all covariates ***X***, i.e., *Y* ∐ *R* ***X*** and the effects of mediator ***M*** on outcome *Y* are unconfounded conditional on the non-manipulable exposure *R* and all covariates ***X***, i.e., *Y* ∐ ***M*** | *R*, ***X***. With these sufficient identifiability assumptions and the models (1)–(2) proposed in the SparseMCMM_HD framework, disparity measures MDM, RDM, and ODM can be further expressed, respectively, as follows (see Section S1 for the detailed derivations):

$$\mathrm{MDM}=\overset{J}{\underset{j=1}{\sum }}\left(\alpha_{Mj}+\alpha_{Cj}\right)\left\{E\left[\log\left(M_{j}\right)|R=1,x\right]-E\left[\log\left(M_{j}\right)|R=0,x\right]\right\}$$

$$\text{RDM}=\alpha_{R}+\alpha_{C}^{T}E[\log(M)|R=0,x]=\alpha_{R}+\overset{J}{\underset{j=1}{\sum }}\alpha_{Cj}E\left[\log\left(M_{j}\right)|R=0,x\right],$$

and

$$\begin{array}{l} \text{ODM}=\text{MDM}+\text{RDM}\\ \;\;=\alpha_{R}+\overset{J}{\underset{j=1}{\sum }}\left(\alpha_{Mj}+\alpha_{Cj}\right)E\left[\log\left(M_{j}\right)|R=1,x\right]-\overset{J}{\underset{j=1}{\sum }}\alpha_{Mj}E\left[\log\left(M_{j}\right)|R=0,x\right], \end{array}$$

where $E\left[\log\left(M_{j}\right)|R=r,x\right]=\text{ψ}\left[\gamma_{j}(R=r,x)\right]-\text{ψ}\left[\sum_{m=1}^{J}\gamma_{m}(R=r,x)\right],\gamma_{j}(R=r,x)=\exp\left(\beta_{0j}+\beta_{Rj}r+\beta_{Xj}^{T}x\right),r=0\;\text{or}\;1,\;\text{and}\;\text{ψ}(\cdot )=\frac{d}{dx}\ln(\text{Γ}(x))$ is the digamma function, with given covariates ***x***.

Note that these mathematical expressions of RDM and MDM are the same as the formulas of causal direct effect of treatment and mediation effect through microbiome correspondingly on the outcome in the typical three-factor causal design based on the traditional causal mediation inference, developed in our SparseMCMM [12]. Analogous to ME in SparseMCMM, MDM is the summation of individual mediation effects from each taxon *MDM_j_*: $\mathrm{MDM}:=\sum_{j=1}^{J}{\mathrm{MDM}}_{j}$ and *MDM_j_* = (*α*_*Mj*_ + *α*_*Cj*_){*E*[log(*M_j_*)|*R* = 1, ***x***] − *E*[log(*M_j_*)|*R* = 0, ***x***]}. *MDM_j_* thus is non-zero only when both the *j*th microbial effect on the outcome and the exposure effect on the *j*th taxon are not zero. Therefore, SparseMCMM_HD illuminates the mediating role of microbiome in the health disparity of outcome, and quantifies the manipulable disparity for overall microbiome community and for each specific taxon, respectively.

#### Parameter estimation.

Note that in [12], we have demonstrated the excellent performance of SparseMCMM in terms of estimation by extensive simulations and real data analysis in various scenarios. Thus for SparseMCMM_HD, we directly employ the same two-step procedure to estimate the regression parameters in models (1)–(2) to obtain the estimated RDM, MDM, *MDM_j_* for each taxon, and ODM. Furthermore, SparseMCMM_HD has the full capability to perform variable selection to select the signature causal microbes that play mediating roles in the disparity of the continuous outcome with regularization strategies. Specifically, L_1_ norm and group-lasso penalties are incorporated for variable selection meanwhile addressing the heredity condition.

#### Hypothesis tests for manipulable disparity.

Similarly, we employ the hypothesis tests for mediation effects in SparseMCMM to examine whether microbiome has any mediation effect on the disparity in an outcome, at both community and taxon levels. Specifically, regarding the null hypothesis of no manipulable disparity *H*_0_: MDM = 0, the first test statistic is defined as $\mathrm{OMD}=\hat{\mathrm{MDM}}$, the estimator of the manipulable disparity. Meanwhile, we consider another null hypothesis, *H*_0_: *MDM_j_* = 0, ∀ *j* ∈ {1,⋯,*J*} and define the second test statistic as $\mathrm{CMD}=\sum_{j=1}^{J}{\hat{\mathrm{MDM}}}_{J}^{2}$, the summation of the squared estimators of individual mediation effects across all taxa. Permutation procedure is employed to assess the significance of these two test statistics. This provides a mechanism to check whether microbiome has any impact on health disparity that could be potentially eliminated through microbiome.

#### Implementation.

The simulation evaluation results regarding the estimation and testing of SparseMCMM [12] are applicable to SparseMCMM_HD framework. Therefore, the proposed SparseMCMM_HD is a validated analytic tool to illuminate the mediating role of microbiome in the disparity of outcome, and quantifies the manipulable disparity for overall microbiome community and for each specific taxon, respectively. In practice, we perform both parameter estimation and hypothesis testing using the analytical procedures in the SparseMCMM package and illustrate the proposed SparseMCMM_HD pipeline through an interactive web app (https://chanw0.shinyapps.io/sparsemcmm_hd/).

### Control for confounding covariates

Due to the non-manipulable nature of the exposure in health disparity research, in principle, it is impossible to design a randomized trial on the exposure of interest to eliminate the potential confounding effect on the interested causal pathway. Many studies on health disparity are observational and usually include significant degrees of confounding, due to factors such as lifestyle, health status, and disease history. We want to emphasize that it is a necessary step to control for confounding covariates while utilizing the proposed SparseMCMM_HD to estimate RDM, MDM, and ODM in a typical observational study. Specifically, we propose to perform propensity score matching (PSM) [31], which is a commonly used method in biomedical research to create a balanced covariate distribution between two groups, to control confounding covariates in our applications (see Section S2). Standardized mean difference (SMD) is used to evaluate the balance of the covariate distributions between groups. A SMD that is less than 0.1 indicates a balanced distribution [32]. The matched data will then be used to quantify RDM, MDM, and ODM, and examine whether the microbiome could reduce the health disparity between two non-manipulable exposure groups. The control for confounding covariates procedure has been included as a preprocessing step in the proposed SparseMCMM_HD analytic pipeline.

### curatedMetagenomicDataV3.4.2

The curatedMetagenomicData 3.4.2 package [28] provides a curated human microbiome meta dataset aggregated from 86 shotgun sequencing cohorts in 6 body sites. The raw sequencing data were processed using the same bioinformatics protocol and pipelines. Each sample has 6 types of data available including gene family, marker abundance, marker presence, pathway abundance, pathway coverage, and taxonomic (relative) abundance. The taxonomic abundance was calculated with MetaPhlAn3, and metabolic functional potential was calculated with HUMAnN3. The manually curated clinical and phenotypic metadata are available as well. More details can be found in the curatedMetagenomicData package document [28]. Here we focus on healthy subjects to explore the relationship among region, microbiome, and BMI. Specifically, we chose subjects from all cohorts based on the following inclusion criteria: 1) healthy status; 2) no missing values in BMI, gender, and age; 3) age ≥ 18; 4) no pregnant; 5) currently no antibiotic use; 6) currently no alcohol consumption; 7) no smoking; and 8) fecal sample with more than 1,250 sample reads. In addition, when multiple samples available for a subject, we randomly selected one sample. Overall, we identified 4,868 healthy adults from different regions. Here we further focus on three regional groups which have large sample sizes: China (n=570), United States (USA; n=350), and United Kingdom (UK; n=1019) for the analysis in the main text. Specifically, we conducted two comparison studies: China-USA and China-UK comparisons to investigate the regional difference of BMI in the China group compared to the USA and UK groups, respectively.

### American Gut Project

The AGP project is a crowd-sourcing citizen science cohort to describe the comprehensive characterization of human gut microbiota and to identify factors being linked to human microbiota. The AGP includes 16S rRNA V4 gene sequences from more than 8,000 fecal samples using standard pipelines, and host metadata. Detailed descriptions can be found in Liu et al. and Hu et al. [1, 33]. Our primary investigation is on the disparity of BMI between Asian or Pacific Islander (API) and non-Hispanic Caucasian adults. We selected a subset of the AGP data based on the following inclusion criteria: 1) USA resident; 2) Asian or Pacific Islander or Caucasian race; 3) no missing values in gender, age, and BMI; 4) age ≥ 18; 5) 80 ≥ BMI; 6) 210cm ≥ height ≥ 80cm; 7) 200kg ≥ weight ≥ 35kg; 8) fecal sample with more than 1,250 sample reads; 9) not duplicate sample; and 10) no self-reported history of inflammatory bowel disease, diabetes, or antibiotic use in the past year. The subjects are filtered out when the reported BMIs are not consistent with the calculated BMI based on the reported heights and weights, i.e. (|BMI_reported_ − BMI_calculated_|/BMI_calculated_ > 5%). A dataset with 130 API and 2,263 Caucasian adults then is used in this paper (Figure S1a).

### Statistical Analysis

Data pre-processing and PSM were conducted in three BMI studies. Specifically, for the China-USA and China-UK comparisons, we performed PSM with the parameters described in Section S2 to control for age and gender. For the API-Caucasian comparison, as the AGP includes more than 400 covariates that were collected through self-reported surveys, we first implemented several pre-processing steps to prepare the self-reported covariates for the subsequent analysis, including cleaning up the inconsistent definition of variables, and collapsing the sparse categorical variables into fewer and less sparse categories. Details are provided in Section S3. Forty-four covariates were retained for PSM. We performed univariate linear regressions to identify the potential confounding variables for the relationship among race, microbiome, and BMI. Twenty-three covariates (p-value ≤ 0.05; Figure S1b) were identified as confounders that need to be controlled further based on PSM.

With the matched data, alpha (Observed, Shannon, and Simpson indices) and beta diversities (Bray–Curtis dissimilarity and Jensen–Shannon divergence) were used to estimate microbial community-level diversity. T tests were used for group comparisons of BMI and alpha diversity. Permutational multivariate analysis of variance (PERMANOVA) [34] was used to assess group difference of beta diversity. We performed the proposed SparseMCMM_HD framework at the species rank (Section S4) to quantify RDM, MDM, and ODM, and examine whether the microbiome could explain the health disparity between two non-manipulable exposure groups. The proposed SparseMCMM_HD pipeline was implemented through an interactive web app (https://chanw0.shinyapps.io/sparsemcmm_hd/) for easy exploration.

## Results

### Results for curatedMetagenomicDataV3.4.2

#### Matched datasets.

**Figure 2.**
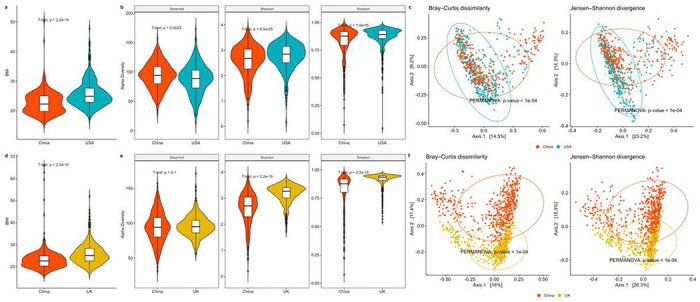
Association analyses in two matched datasets from the curatedMetagenomicData package [28]. a Violin plots of BMI in matched Chinese vs. USA subjects. b Violin plots of alpha diversities (Observed, Shannon, and Simpson indices) in matched Chinese vs. USA samples. c PCoA plots using Bray–Curtis dissimilarity and Jensen–Shannon divergence in matched Chinese and USA samples. d Violin plots of BMI in matched Chinese vs. UK subjects. e Violin plots of alpha diversities (Observed, Shannon, and Simpson indices) in matched Chinese and UK samples. f PCoA plots using Bray–Curtis dissimilarity and Jensen–Shannon divergence in matched Chinese vs. UK samples.

With the healthy adults included in the China-USA and China-UK comparisons, we identified 328 matched Chinese-USA subject pairs, and 559 matched Chinese-UK subject pairs, separately. Figures S2 and S3 show that both matched datasets have comparable propensity scores. The SMDs decrease dramatically on the matched subjects (SMD=0.036 and 0.033), from using all subjects (SMD=0.302 and 0.470) in both China-USA and China-UK datasets. This indicates that PSM has effectively evened the distribution of confounders between two exposure groups in our studies and practically eliminated or controlled the influence of the confounders. In the well-matched datasets, the China group still has significantly lower average BMIs compared to the matched USA (mean [standard deviation]: 22.64 [3.77] vs. 25.77 [4.56]) and the matched UK (22.98 [4.48] vs. 25.77 [4.79]) groups (Figure 2a and 2d).

#### Community level results.

The Chinese group has distinctive microbial community diversities, compared to the matched USA or UK group. For alpha diversity, samples from China have lower Shannon and Simpson diversities and a higher observed diversity than the matched USA or UK samples (Figure 2b and 2e). For beta diversity, Bray-Curtis dissimilarity and Jensen-Shannon divergence both indicate that the Chinese group is significantly different in community structure from the matched USA or UK groups (PERMANOVA [34] all p-values < 1.0 × 10^−4^. Figure 2c and 2f).

#### Taxon-level analysis.

**Figure 3.**
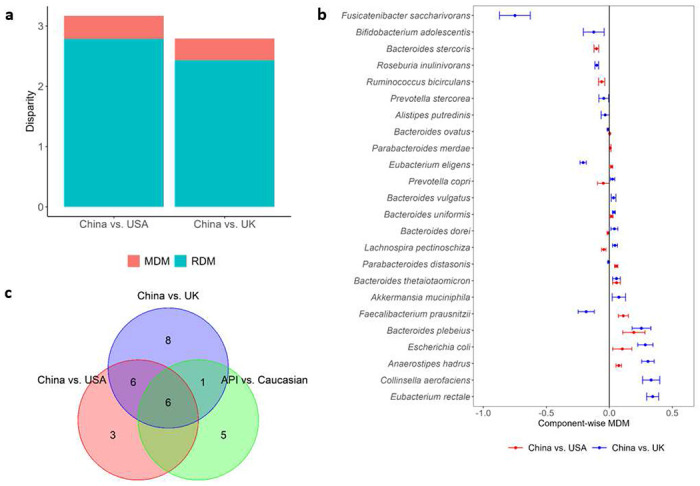
Health disparity analyses in two matched datasets from the curatedMetagenomicData package [28]. a Manipulable disparity measure (MDM) and residual disparity measure (RDM) of BMI in the China-USA comparison and China-UK comparison, respectively. b Component-wise point and 95% CI estimates of *MDM_j_* for the identified species that have mediation effects on the differences of BMI between matched Chinese vs. USA subjects and between matched Chinese vs. UK subjects, respectively. 95% CI estimates of *MDM_j_* were calculated by bootstrapping procedure, and the number of bootstrapping is 50. c Venn diagram to show the relationship of the species playing mediation effects in the disparity of BMI among China-USA, China-UK, and API-Caucasian comparisons. API: Asian or Pacific Islander.

After implementing the filtering criteria described in Section S4, 25 species remained in both matched datasets (China vs. USA and China vs. UK). The testing results for OMD and CMD show that the overall and component-wise MDMs through microbiome are significant in both data sets for regional differences in BMI (all p-values<0.001 based on 1,000 permutations). Figure 3a shows that the ODM of BMI are 3.17 and 2.79, respectively, for the matched Chinese and USA subjects, and the matched Chinese and UK subjects; the corresponding MDMs due to microbiome are 0.38 and 0.36. These results suggest that 11.99% and 12.90% of the disparity in BMI between the Chinese and matched USA and UK groups, respectively, would be eliminated if the between-group microbiome profiles were equalized.

**Figure 4.**
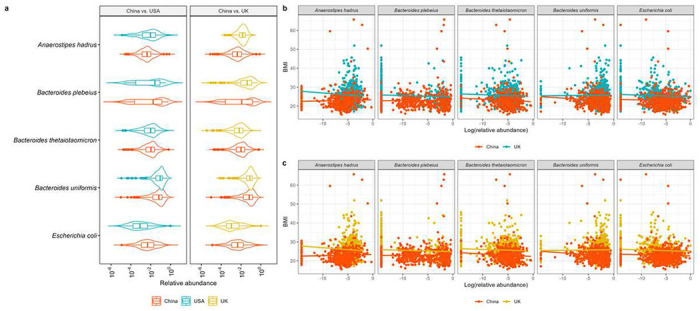
Five species who play positive mediation roles in the disparity of BMI in both China-USA and China-UK comparisons. a Violin plots illustrating the relative abundances of these five identified species in the matched Chinese and USA samples, and the matched Chinese and UK samples, respectively. b Scatterplots of BMI and the relative abundances of these five identified species in the matched Chinese and USA subjects, and the matched Chinese and UK subjects, respectively.

**Figure 5.**
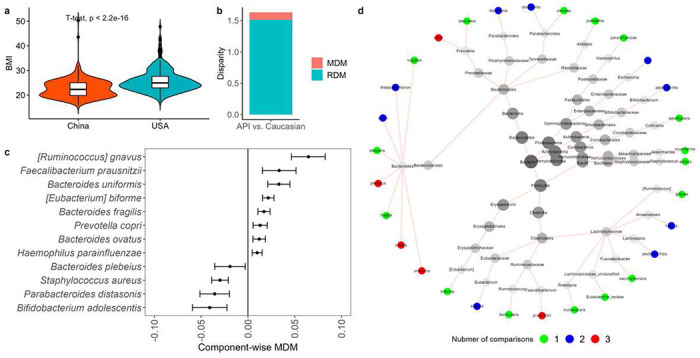
Health disparity analyses in the matched APIs and Caucasians from the AGP dataset. a Violin plots of BMI in the matched APIs and Caucasians from the AGP dataset. b MDM and RDM of BMI in the API- Caucasian comparison. c Component-wise point and 95% CI estimates of *MDM_j_* for the identified species that have mediation effects on the differences of BMI between matched APIs and Caucasians from the AGP dataset. 95% CI estimates of *MDM_j_* were calculated by bootstrapping procedure, and the number of bootstrapping is 50. d The taxonomic relationship of the species playing mediation effects in the disparity of BMI among China-USA, China-UK, and API-Caucasian comparisons. The tree figure was generated by Metacoder [65]. From the outer to the center, taxonomic ranks are species, genus, family, order, class, phylum, and kingdom (Bacteria), respectively. For each species, color represents the number of comparisons that identify it among China-USA, China-UK, and API-Caucasian comparisons. APIs: Asian or Pacific Islanders.

Significant CMD testing results show that there is at least one species playing a mediating role in the disparity of BMI between Chinese and USA subjects, and Chinese and UK subjects. Figure 3b reports 15 species and 21 species further identified by SparseMCMM_HD, with the point and 95% confidence interval (CI) estimates for their mediation effects on the regional differences of BMI between China and USA, and between China and UK, respectively. Among the twelve overlapping species identified in both matched datasets (Figure 3b and 3c), five species— *Anaerostipes hadrus, Bacteroides plebeius, Bacteroides thetaiotaomicron, Bacteroides uniformis*, and *Escherichia coli*—play consistent positive mediating roles in regional disparity in BMI for Chinese compared to USA subjects, and for Chinese compared to UK subjects. The relative evaluation of these five species in terms of their relative abundances (Figure 4a) and their associations with BMI (Figure 4b) are quite similar between two independent studies: China-USA comparison and China-UK comparison, which validates their mediating roles in the regional disparity on BMI. Confirming with the published studies, *B. plebeius, B. thetaiotaomicron*, and *B. uniformis* belong to the same genus *Bacteroides*, and all play important roles in human metabolism and have been linked with diet-induced obesity, by improving whole-body glucose disposal, promoting lipid digestion and absorption, and degrading host-derived carbohydrates [35–38]. *B. thetaiotaomicron* also possesses glycine lipid biosynthesis pathway (Figure S4). *A. hadrus*, and *E. coli* also have been reported by multiple studies that they contribute to or are associated with the BMI or obesity [39–41]. On the other hand, 12 species play mediating roles in BMI but with the opposite directions between China-USA comparison and China-UK comparison, that reflects the distinguishing characteristics between USA and UK (Figure S5). This is not surprising considering the microbial profile is inherently dynamic and racially or geographically specific. Moreover, there are three and nine unique species identified in the China-USA and China-UK comparisons respectively (Figures S6 and S7). Most of these study-specific species have been reported being associated with BMI, obesity or metabolic disorders [41–50]. Notably, *Anaerostipes hadrus, Fusicatenibacter saccharivorans, Lachnospira pectinoschiza*, and *Roseburia inulinivorans* belong to family *Lachnospiraceae* (Figure 5d), which is related to metabolic syndrome and obesity and whose controversial role has been discussed across different studies [51].

### Results for AGP

#### Matched dataset.

After performing PSM, as described in Section S2, 98 Caucasians and 98 APIs are matched. Figures S8 and S9 show that the matched Caucasians and APIs have very similar propensity scores (SMD=0.005 for the matched subjects vs. SMD=1.033 for the raw subjects), indicating that the confounding effects are well controlled. With this well-matched dataset, Figure 5a shows that the Caucasian group has a significantly higher BMI (23.96 [3.92]), compared to the API group (22.38 [3.59]), as observed in the other studies [52, 53].

#### Community level results.

Caucasians and APIs have distinct microbial profiles in terms of community diversity. For alpha diversity, Caucasians have higher microbial richness and evenness as measured by Observed, Shannon, and Simpson diversities (p-value = 3.1 × 10^−5^, 1.5 × 10^−4^, and 3.9 × 10^−3^, respectively. Figure S10a). For Beta diversity, Bray-Curtis dissimilarity and Jensen-Shannon divergence both show that Caucasian samples have different community structures compared to API samples (PERMANOVA p-value=0.0036 and 0.0012, respectively. Figure S10b).

#### Taxon-level analysis.

The above community level results indicate that the microbiome may play a mediating role in the racial diversity of BMI. To investigate this assumption, we perform the proposed SparseMCMM_HD on this matched dataset. With the filtering criteria described in Section S4, 28 species are included in the following taxon-level analysis.

We found that the ODM of BMI between Caucasians and APIs is 1.63 (Figure 5b). Microbiome plays a significant role in mediating the racial disparity of BMI indicated by the test results of both OMD (p-value=0.038) and CMD (p-value=0.048). The microbial manipulable disparity measure MDM is 0.12. This suggests that the difference of microbiome profiles contributes to 7.4% of ODM, which would be eliminated if the microbiome profiles between the Caucasians and APIs were identical.

We further identified 12 species playing mediating roles in the racial disparity of BMI between the Caucasians and APIs (Figure 5c). Eight species (*[Ruminococcus] gnavus, Faecalibacterium prausnitzii, Bacteroides uniformis, [Eubacterium] biforme, Bacteroides fragilis, Prevotella copri, Bacteroides ovatus, Haemophilus parainfluenzae*) mediate positively on the racial disparity of BMI, meanwhile, four species (*Bifidobacterium adolescentis, Bacteroides plebeius, Parabacteroides distasonis, Staphylococcus aureus*) play negative mediating roles. Remarkably, there are six common species *B. ovatus, B. plebeius, B. uniformis, B. adolescentis, F. prausnitzii, P. distasonis*, and *P. copri* identified by both comparisons: China-USA and China-UK illustrated in the previous subsection (Figure 5d). Literature reveals that all identified species are associated with the BMI or obesity [41–49].

Collectively, the findings in the matched China vs. USA, China vs. UK, and API vs. Caucasian datasets show that the microbiome is an important mediator in the regional or racial disparity of BMI and they substantially shed light on how to reduce the disparity of BMI. The identified microbial agents can be used as the potential therapeutic target for the treatment based on microbiota modulation in the future.

## Discussion

The emerging evidence highlights the potential of microbiome in understanding health disparity. In this paper, we proposed a mediation analytical framework, SparseMCMM_HD, to investigate the microbiome’s role in health disparity. Considering a health disparity framework with three components: non-manipulable exposure (e.g. race or region), microbiome as mediator, and outcome, the proposed SparseMCMM_HD deciphers the overall health disparity of the non-manipulable exposure on the outcome into two components: MDM that would be eliminated by equalizing microbiome profiles and RDM that would remain and could not be explained through microbiome. Remarkably, MDM paves a viable path towards reduction of health disparity with microbial modulation. Similar to SparseMCMM, SparseMCMM_HD can be used to identify the signature causal microbes and examine whether the overall or component-wise MDM is significantly non-zero.

It is vital to control confounding effects beforehand in the real data analysis to satisfy the identifiability assumptions of the proposed SparseMCMM_HD. In three BMI applications, we first employed PSM to remove the confounding effects by selecting matched subsets in which the distributions of confounders were notably comparable between two exposure groups, and then performed the proposed SparseMCMM_HD framework. The utilization of SparseMCMM_HD in two datasets, the curatedMetagenomicData 3.4.2 package and the AGP dataset, depicts an explicit causal path among region or race, microbiome, and BMI. These findings confirm not only that microbiome is differentially distributed across races or regions, but also that the differential microbiome profile contributes to the disparities in BMI across races or regions. The identified microbial signatures potentially aid in developing personalized medication or nutrition to reduce obesity disparity.

It is not surprising that the proportion of disparities in BMI explained by the microbiome profiles is not large (~10%) in all three applications, due to the heritable and polygenic nature of BMI [54, 55]. Further investigations to integrate the microbiome profile and genetic factors are necessary to better understand disparity in BMI. However, we here emphasize that the proposed SparseMCMM_HD is a rigorous and validated causal mediation framework and has preeminent potential to identify the microbiome’s roles in much broader health disparity studies.

Recently, several other microbial mediation methods have been proposed, such as CMM [56], MedTest [57], Zhang, et al. [58], LDM-med [59], and MarZIC [60], in a typical three-factor (manipulable exposure, microbiome as mediator, and outcome) study design. Considering distinct model assumptions and characteristics, a few recent benchmark studies [12, 56–60] show that there is no method performing consistently and accurately better than others in all circumstances. However, since the assumptions for model identification in health disparity are weaker than those for the causal mediation effects in the manipulable exposure-mediator-outcome framework [23], it is expected that the idea of how the proposed SparseMCMM_HD framework rigorously defines, quantifies, and tests health disparity measures as an extension of SparseMCMM [12] can provide insight into extending these available mediation models to investigate the microbiome’s role in health disparity. Then, a useful path forward will be to mutually employ these multiple and complimentary methods to better characterize the microbiome’s role in health disparity by capitalizing their distinct assumptions and strengths.

Our study has several limitations. First, similar to discussions in SparseMCMM [12], SparseMCMM_HD takes microbiome data at a fixed time point into the proposed frame and is limited to accommodate the dynamic nature of microbiome. Second, the proposed SparseMCMM_HD currently deals with disparity in a continuous outcome. Given the fact that multiple binary or categorical outcomes are disproportionately prevalent across races or regions [61–63], it will be worthwhile to extend the current framework to handle categorical outcomes. Third, microbiome studies typically characterize both taxonomic and functional profiles of microbial communities. Functional profile is generally thought to be more closely linked with human health and disease. Identifying the role of microbiome in terms of gene function in health disparity is of high practical value [64].

## Conclusions

This paper elucidates the role of microbiome in health disparity by providing a causal mediation analytic framework for investigating the relationship among race or region, microbiome, and outcome under the counterfactual framework. The proposed SparseMCMM_HD framework is a useful tool to investigate the underlying biological mechanism of health disparity and disentangles the substantial contributions of microbiome to health disparity. The applications of SparseMCMM_HD in the disparity of BMI across races and regions uncover the microbial mediating roles in reducing the disparities of BMI and improving health equality.

## Data Availability

All relevant datasets are publicly available. The data used in investigations of the regional difference of BMI in the China group compared to the United States (USA) and United Kingdom (UK) groups can be downloaded from the curatedMetagenomicData 3.4.2 package [28]. The data used in investigations of the racial difference in BMI between Caucasians and Asian or Pacific Islanders are from the American Gut Project. Their raw data and metadata are publicly available on the FTP website (ftp://ftp.microbio.me/AmericanGut/). Version 07/29/2016 is used in our analyses. SparseMCMM R package is available at https://github.com/chanw0/SparseMCMM. The interactive web app for the proposed SparseMCMM_HD framework is available at https://chanw0.shinyapps.io/sparsemcmm_hd/.

## List of abbreviations

AGP: American gut project
API: Asian or Pacific Islander
BMI: body mass index
MDM: manipulable disparity measure
ODM: overall disparity measure
PERMANOVA: permutational multivariate analysis of variance
PSM: propensity score matching
RDM: residual disparity measure
SparseMCMM: sparse microbial causal mediation model
SparseMCMM_HD: SparseMCMM for health disparity
SMD: standardized mean difference
UK: United Kingdom
USA: United States
